# Evaluating Baseline Sleep and Feedback on The ABCs of Zzz, a Sleep Program in Black Churches

**DOI:** 10.1093/geroni/igaf122.1915

**Published:** 2025-12-31

**Authors:** Glenna Brewster, Daum Chung, Miranda McPhillips, Elliane Irani, Fayron Epps

**Affiliations:** Emory University, Atlanta, Georgia, United States; Emory University, Atlanta, Georgia, United States; Villanova University, Villanova, Pennsylvania, United States; Case Western Reserve University, Cleveland, Ohio, United States; UT Health San Antonio, San Antonio, Texas, United States

## Abstract

Sleep disparities disproportionately affect Black/African American communities, with high rates of insomnia and poor sleep quality often going unaddressed. The ABCs of Zzz is a four-hour, in-person sleep education program co-developed with church ambassadors and a community advisory board. The program was delivered at Alter-affiliated, predominantly Black/African American churches in Georgia. This study presents baseline sleep characteristics of participants and their initial feedback on the program. Participants completed demographic information and validated sleep measures: Insomnia Severity Index (ISI), Pittsburgh Sleep Quality Index (PSQI), and Dysfunctional Beliefs and Attitudes about Sleep (DBAS). Following the session, participants provided feedback on program satisfaction. Descriptive statistics were used for analysis. The sample (N = 134) was predominantly female (77.4%) and Black/African American (97.0%). Participants ranged in age from 18 to 91 years (M = 62.77, SD = 13.05), with half of the participants being older than 64 years. Participants had mean baseline ISI score of 9.52 (SD = 6.80), indicating mild to moderate insomnia symptoms. The mean PSQI score was 9.44 (SD = 4.21), suggesting poor sleep quality. DBAS scores averaged 4.12 (SD = 2.31), suggesting flawed sleep beliefs. Session feedback was positive;100% of participants would refer a friend or family member; 84.1% were “very satisfied” with the content; and 98.9% agreed the session met their expectations. Baseline data highlight significant sleep challenges among participants from predominantly Black/African American churches, emphasizing the need for targeted sleep interventions in this community. The overwhelmingly positive feedback underscores the value of community-driven sleep education programs.

